# Proinflammatory mediators, TNFα, IFNγ, and thrombin, directly induce lymphatic capillary tube regression

**DOI:** 10.3389/fcell.2022.937982

**Published:** 2022-07-19

**Authors:** Scott S. Kemp, Marlena R. Penn, Gretchen M. Koller, Courtney T. Griffin, George E. Davis

**Affiliations:** ^1^ Department of Molecular Pharmacology and Physiology, Morsani College of Medicine, University of South Florida School of Medicine, Tampa, FL, United States; ^2^ Cardiovascular Biology Research Program, Oklahoma Medical Research Foundation, Oklahoma City, OK, United States

**Keywords:** lymphatic endothelial cells, capillaries, capillary regression, proinflammatory mediators, tumor necrosis factor, interferon gamma, thrombin, pharmacological rescue of lymphatic capillary regression

## Abstract

In this work, we sought to investigate the direct effects of proinflammatory mediators on lymphatic endothelial cell (LEC) capillaries and whether they might induce regression. Our laboratory has developed novel *in-vitro*, serum-free, lymphatic tubulogenesis assay models whereby human LEC tube networks readily form in either three-dimensional collagen or fibrin matrices. These systems were initially conceptualized in the hopes of better understanding the influence of proinflammatory mediators on LEC capillaries. In this work, we have screened and identified proinflammatory mediators that cause regression of LEC tube networks, the most potent of which is TNFα (tumor necrosis factor alpha), followed by IFNγ (interferon gamma) and thrombin. When these mediators were combined, even greater and more rapid lymphatic capillary regression occurred. Surprisingly, IL-1β (interleukin-1 beta), one of the most potent and pathologic cytokines known, had no regressive effect on these tube networks. Finally, we identified new pharmacological drug combinations capable of rescuing LEC capillaries from regression in response to the potent combination of TNFα, IFNγ, and thrombin. We speculate that protecting lymphatic capillaries from regression may be an important step toward mitigating a wide variety of acute and chronic disease states, as lymphatics are believed to clear both proinflammatory cells and mediators from inflamed and damaged tissue beds. Overall, these studies identify key proinflammatory mediators, including TNFα, IFNγ, and thrombin, that induce regression of LEC tube networks, as well as identify potential therapeutic agents to diminish LEC capillary regression responses.

## Introduction

Lymphatic vessels are often defined by three key functions: 1. absorption of lipids (and fat-soluble vitamins), 2. immune surveillance, and 3. uptake of tissue fluids along with leukocytes. It is important to note that tissue fluid absorption is not limited to water, but also to a variety of proteins and other molecules, including growth factors, small molecules, peptides, cytokines, hormones, and nutrients (Adams and Alitalo, 2007; Wang and Oliver, 2010; Alitalo, 2011; Randolph et al., 2017; Breslin et al., 2018; Petrova and Koh, 2018; Cifarelli and Eichmann, 2019; Oliver et al., 2020; Stritt et al., 2021). Healthy lymphatic systems facilitate the resolution of inflammation in damaged tissues, while dysfunctional lymphatic systems are believed to cause a buildup of proinflammatory leukocytes and mediators, which can further lead to edema and other pathological manifestations (Schwager and Detmar, 2019). Lymphatic dysfunction has gained increasing attention as it is directly linked to lymphedema and cancer (Skobe et al., 2001; Padera et al., 2002; Hoshida et al., 2006; Cormier et al., 2010; Chakraborty et al., 2013), but also to many other disease states including inflammatory bowel diseases such as Crohn’s disease, rheumatoid arthritis, various skin inflammatory conditions, atherosclerosis, obesity, type 2 diabetes, dry eye disease, Alzheimer’s disease and myocardial infarction (Paavonen et al., 2002; Wauke et al., 2002; Huh et al., 2005; Wu et al., 2006; Lim et al., 2009; Goyal et al., 2010; Iliff et al., 2012; Haemmerle et al., 2013; Martel et al., 2013; Sawane et al., 2013; Carlson, 2014; Bouta et al., 2015; Scallan et al., 2015; Tarasoff-Conway et al., 2015; Kanapathy et al., 2016; Vieira et al., 2018; Zhang et al., 2018; Cifarelli and Eichmann, 2019). Whereas lymphedema is often attributed to lymphatic valve dysfunction within collecting vessels, we speculate that many of these diseases could have lymphatic capillary deficiencies as well. Lymphatic capillaries are unique in their morphology, having a discontinuous distribution of VE-cadherin in their junctions, as well as lacking both a continuous basement membrane and mural cell (pericyte) coverage (Dejana and Orsenigo, 2013; Breslin et al., 2018; Zhang et al., 2020). These properties facilitate the ability of these specialized vessels to take up cells and fluid components for tissue homeostasis as well as the resolution of tissue injury states. Additionally, lymphatic capillaries are known to have larger diameters than their blood capillary counterparts (Schmid-Schonbein, 1990; Pepper and Skobe, 2003; Baluk et al., 2007). The link between pericyte coverage and basement membrane deposition has been well documented in blood capillaries, thus it makes sense that the absence of pericyte coverage would result in a lack of basement membrane deposition around these vessels. Previous studies have demonstrated increased blood capillary widths when pericytes are blocked or not present due to lack of basement membranes. This difference may explain the increased tube diameters observed with lymphatics (Stratman et al., 2009; Stratman et al., 2010; Stratman et al., 2017; Bowers et al., 2020; Kemp et al., 2020). Overall, these specialized properties of lymphatic capillaries allow them to clear proinflammatory cells and mediators, which promotes tissue and organismal health by resolving inflammation.

Considerable information is known concerning the impact of proinflammatory mediators on the blood vasculature including increased vascular permeability, leukocyte adhesion and transmigration, coagulation, platelet adhesion and aggregation (Ridker et al., 2000; Sainson et al., 2008; Arroyo and Iruela-Arispe, 2010; Rigor et al., 2012; Rigor et al., 2013; De Caterina et al., 2016; Mitoma et al., 2018; Jackson et al., 2019; Ridker, 2019). Recent work identified the direct effects of proinflammatory mediators on blood endothelial cell (BEC) capillary tube networks (Koller et al., 2020). Several mediators were found to induce BEC capillary regression. The most potent combination included IL-1β (interleukin-1 beta), TNFα (tumor necrosis factor alpha), and thrombin (Koller et al., 2020). These molecules already represent therapeutic targets in various treatment regimens including atherosclerosis, rheumatoid arthritis, Crohn’s disease, psoriasis, deep vein thrombosis, pulmonary embolism, and malignant cancers (Arroyo and Iruela-Arispe, 2010; Ridker et al., 2017a; Ridker et al., 2017b; Mitoma et al., 2018; Ridker et al., 2018; Jackson et al., 2019; Libby and Everett, 2019; Ridker, 2019). Regression of blood capillaries has been identified as pathogenically linked to hypertension, renal ischemic injury, aging, and neurodegeneration, and may be a critical pathogenic feature of many other major human diseases (Noon et al., 1997; Basile, 2004; Battegay et al., 2007; Sim et al., 2014; Tibirica et al., 2015; Manukhina et al., 2016; Hinkel et al., 2017; Mejia-Renteria et al., 2019; Yeung et al., 2019; Zeng and Chen, 2019). It is becoming increasingly evident that we need to better understand the cell biology of both lymphatic and blood capillaries, as these two types of capillary networks fundamentally support and affect each other. Disease states in one vascular system are likely to cross-over and affect the functional status of the other vasculature.

In this work, we set out to determine the direct effect of proinflammatory mediators on lymphatic capillary tube networks. Using a highly defined culture system whereby human lymphatic endothelial cells (LECs) form interconnecting networks of tubes in either three-dimensional (3D) collagen or fibrin matrices, we were able to assess the direct effects of proinflammatory mediators, added individually or in combination after LEC tube networks have formed. We found that several proinflammatory mediators induced regression of LEC tube networks in either collagen or fibrin matrices, with the three most potent factors being TNFα, IFNγ (interferon gamma) and thrombin. Even greater regression of lymphatic capillary networks occurred when these mediators were added in combination. In contrast, IL-1β, a potent inducer of blood capillary network regression (Koller et al., 2020), did not induce regression of lymphatic capillaries in our defined system. Finally, we have identified combinations of pharmacological agents that rescue lymphatic regression in response to the combination of TNFα, IFNγ, and thrombin. Overall, this work identifies proinflammatory molecules that cause LEC tube network regression, and we propose that lymphatic capillary regression will increasingly be implicated as an important pathogenic feature in a variety of disease states. Furthermore, our work has identified ways to pharmacologically mitigate LEC tube network regression and our novel assay model allows for further elucidation of the biology of LECs, as well as molecular controls of lymphatic tubulogenesis and stabilization in 3D matrix environments.

## Materials and methods

### Materials

The materials for this study are listed in Supplementary Table S1.

### Cell culture

Both human dermal lymphatic ECs (used from passages 3–9) and HUVECs (used from passages 3–6) were grown on gelatin-coated flasks, in CO_2_ cell culture incubators, which were set to 5% CO_2_ and a temperature of 37°C. In-house feeding media, colloquially referred to as ‘Super media,’ was prepared using 20% fetal bovine serum, gentamicin, amphotericin B, heparin sodium salt and bovine hypothalamus extract in an M199 solution (Koh et al., 2008).

### Vasculogenic assays (collagen)

LECs (at 2 × 10^6^ cells/mL) were suspended in a 2.5 mg/ml of type I collagen matrix. Gels were pipetted into a 96 (half area) well plates and given 30 min to polymerize and equilibrate. Next, a five-growth factor system consisting of Reduced Serum Supplement II (RSII) containing insulin (Koh et al., 2008), as well as 50 ng/ml of fibroblast growth factor-2 (FGF-2), 40 ng/ml of stem-cell factor (SCF), 40 ng/ml of stromal cell-derived factor-1 alpha (SDF-1α), and 40 ng/ml of interleukin-3 (IL-3) were added to M199) was placed on top of polymerized gel as a feeding media (Stratman et al., 2011). FIST (10 μM forskolin, 100 μM IBMX, 10 μM SB239063, and 10 μM Tubacin) was added to the feeding media for the first 48 h, when specified. After first 48 h, cell media was removed, newly made factor media with any of the following pharmacologic combinations was added (per experimental design): FIST; FISTSB = FIST +25 μM SB415286; FISTch + SB = 10 μM TCS HDAC6 20b (added in place of tubacin); FISTchSB^2^ = FISTSB +10μM SB43154; FISTchSB^2^K = FISTchSB^2^ + 10 μM K02288. Various proinflammatory mediators were also screened and added directly to the feeding media with FIST, unless otherwise specified. Concentrations are listed in the Supplementary Table S1. Assays were then placed back in the incubator. After 24 h, assays were fixed in 3% paraformaldehyde for immunostaining or 3% glutaraldehyde for toluidine blue staining.

### Vasculogenic assays (fibrin)

LECs (at 2 × 10^6^ cells/mL) were suspended in 5 mg/ml of fibrinogen, containing 100 μg/ml of fibronectin. This gel was then added on top of a 1 μL drop of thrombin with a final concentration of 200 ng/ml (including gel and feeding media). Assays were placed in incubator and allowed to polymerize/equilibrate. After 30 min, feeding media was set up in the same way as listed in collagen system, except aprotinin is added at 5 KIU/ml to feeding media, as well as the addition of 10 mM D-glucose.

### Lumen area quantification

After 72 h, experiments were fixed in 3% paraformaldehyde and stained with toluidine blue. ≥4 pictures were taken from predetermined locations per experiment, and ≥5 validating experimental replicates were conducted in total for all quantitative data. Pictures were traced and area was measured using arbitrary units (see “Microscopy and imaging” for microscopes and software used in this process).

### Immunostaining of 3-dimensional cultures

Collagen or fibrin gels were plucked after fixation in 3% paraformaldehyde. Gels were then washed in a Tris-glycine buffer for 1 h. After 1 h, Tris-glycine buffer was removed, and gels were then permeabilized in a 1% Triton-X100 solution for 1 h. Next, the Triton solution was removed and blocking buffer was added. This blocking buffer contained 5% serum, specific to the secondary antibody. After 1 h of blocking, primary antibody was added directly to blocking buffer and incubated overnight in a 4°C fridge. Afterward, primary antibody and blocking buffer were removed. The gel was subsequently rinsed with tris buffer saline (TBS) several times. After washing, fresh blocking buffer was added back, as well as fluorescent secondary antibody, which was added directly into the blocking buffer. This was allowed to incubate for 2 h, at which point blocking buffer and secondary antibody were removed and gels were rinsed and stored in TBS until sample was ready to be imaged.

### Microscopy and imaging

Images for quantifying LEC lumen area were taken on an Olympus CKX41 microscope (Olympus) and imaging software (Olympus). Time-lapse movies of LECs were taken using a DMI6000B microscope with environmental chamber (Leica Microsystems) and controlled using MetaMorph 7.8 software (Molecular Devices). A ×10 objective was used for all movies. Time lapse movies were created by taking an image every 10 min with a monochromatic Hamamatsu ORCA-ER C4742-80 camera (Hamamatsu) over 0–48 and 48–72 h. Using MetaMorph software, these images were compiled into a movie. Tube areas were also evaluated using MetaMorph. Movies were processed, edited, and stabilized using Adobe Creative Cloud: Adobe After Effects (Adobe Systems). All confocal images were taken on a Leica SP8 LIGHTNING White light laser confocal scanning microscope. Confocal images were taken using either a 10X HC PL APO, 0.4NA (numerical aperture), WD (working distance) 2.2 mm or 40X HC PL APO, water immersion, 1.1NA,WD 0.65 mm lenses. Confocal z-stack reconstructions were created by either using LAS X (Leica Microsystems) or Fiji [(Fiji is just) ImageJ].

### Reverse transcription polymerase chain reaction

Per manufacturers’ guidelines, LEC RNA was extracted using Direct-zol RNA miniprep kit. cDNA was synthesized using a AccuScript first-strand cDNA kit with 500 ng of RNA. All primer sequences can be found in the major resources table in Supplementary Material. Fotodyne system (Fotodyne, Inc.) was used to visualize PCR products after they were run on a 1% agarose gel.

### Western Blots

Western blots were performed after gels were plucked from the collagen system and dissolved using a 1.5% sample buffer with 5% beta-mercaptoethanol. After heating at 100°C for 10 min, samples were cooled. Once samples were ready to be used, samples were pipetted into Biorad gels and ran until ladder reached the bottom. Polyvinylidene fluoride or nitrocellulose membranes were used to acquire proteins from the gel, which was subsequently blocked using 3% bovine serum albumin. Then primary antibodies were added directly to blocking buffer. After incubating overnight at 4°C, membranes were washed, and secondary antibodies conjugated with horseradish peroxidase were added in 3% milk. X-ray film was then developed for final western blot images.

### Statistics

Prism 8 (Graphpad) was used for analysis of variance (ANOVA) (ordinary one-way or two-way) with follow up post-hoc Tukey tests. *p* < 0.05 was determined to be the minimum statistical significance. Experiments were performed with ≥5 validating experimental replicates in total.

## Results

### Human lymphatic capillary tube network assembly in 3D collagen and fibrin matrices

**FIGURE 1:**
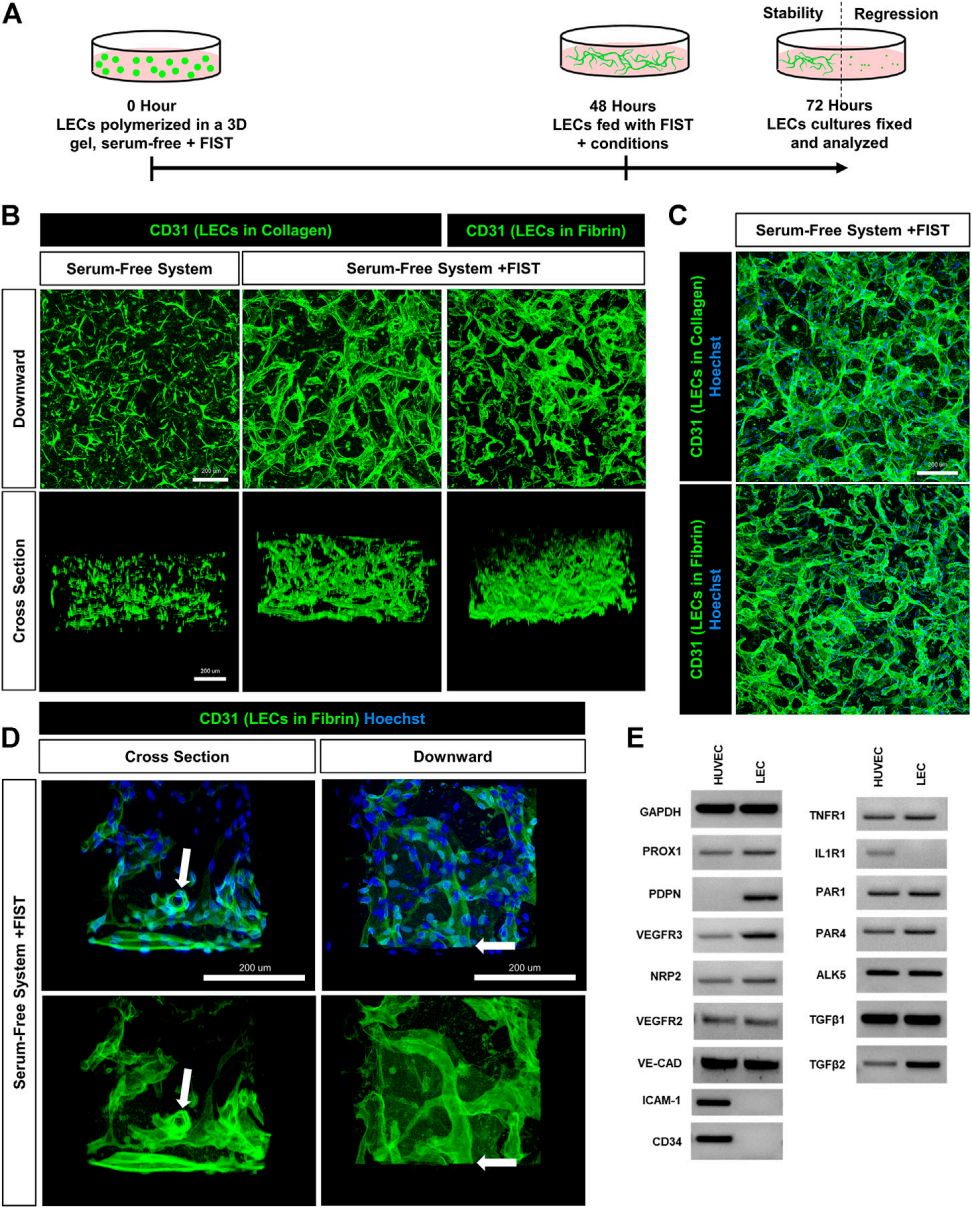
A human lymphatic EC tube network formation model system in 3D collagen or fibrin matrices under serum-free, defined media conditions. **(A)** Schematic timeline highlighting the important events in the assay models. The model utilizes a serum-free, five growth factor system (IL [interleukin]-3, SCF [stem cell factor, SDF [stromal cell-derived factor]-1α, FGF [fibroblast growth factor]-2, and insulin). In addition, a four-drug pharmacologic combination termed FIST (10 μM forskolin, 100 μM IBMX, 10μM SB239063, and 10 μM Tubacin) was added to facilitate the formation of 3-dimensional (3D) lymphatic endothelial cell (LEC) tube networks. **(B,C,D)** LEC tube networks at 72 h. Fixed cultures were stained with anti-CD31 (green) and Hoechst (blue); imaged using confocal z-stack, 3D reconstructions. **(B)** Control LECs in serum-free, collagen gel, but no FIST (left column); LECs + FIST in serum-free, collagen gel (middle column); LECs + FIST in serum-free, fibrin gel (right column); “downward” view of confocal z-stack reconstruction (top row); “cross sectional” view reconstruction (bottom row). **(C)** Additional images of LECs + FIST in serum-free media with nuclei staining in a collagen gel (top) and fibrin gel (bottom). **(D)** LECs + FIST in serum-free media. “Cross sectional” view reconstruction (left column); “Downward” view reconstruction (right column). With nuclei staining (top row); without nuclei staining (bottom row). White arrows point to a lumenized tube that can be observed in cross-section. **(E)** HUVEC (EC) vs. dermal LECs (LEC) mRNA was isolated and RT-PCR analysis was performed. Expression of indicated genes was analyzed. GAPDH (glyceraldehyde-3-Phosphate Dehydrogenase), PROX1 (prospero homeobox 1), PDPN (podoplanin), VEGFR3 (vascular endothelial growth factor receptor 3), NRP2 (neuropilin 2), VEGFR2 (vascular endothelial growth factor receptor 2), VE-CAD (vascular endothelial cadherin), ICAM-1 (intercellular adhesion molecule 1), CD34 (hematopoietic progenitor cell antigen cluster of differentiation 34), TNFR1 (tumor necrosis factor receptor 1), IL1R1 (interleukin 1 receptor type 1), PAR1 and 4 (protease-activated receptor -1 and -4) ALK5 (activin-like kinase 5), TGFβ1 and 2 (transforming growth factor beta-1 and -2). Bars equals 200 μm.

For over 2 decades, our laboratory has developed novel, serum-free, 3D (using both fibrin and collagen matrices) bioassays to understand the fundamental ability of human ECs to form lumens and tube networks and to sprout in 3D extracellular matrices. A key model system that we developed depends on the addition of five growth factors (“Factors”): (IL [interleukin]-3, SCF [stem cell factor], SDF [stromal cell-derived factor]-1α, FGF [fibroblast growth factor]-2, and insulin). When used in combination, these “Factors” induce human BECs to form branched and lumenized tube networks that can be sustained for weeks. This assay platform has been used by our laboratory to study many different processes, including vascular morphogenesis by mimicking vasculogenesis or angiogenic sprouting, lumen and tube assembly, mural cell recruitment and capillary basement membrane deposition, tube stability, tube regression, and vascular malformations (Stratman et al., 2009; Stratman et al., 2010; Stratman et al., 2011; Smith et al., 2013; Bowers et al., 2020; Kemp et al., 2020; Koller et al., 2020; Sun et al., 2022). Addition of the “Factors” to LECs in similar assays yielded cord-like assembly with limited tube formation that appears to disassemble over time in either collagen or fibrin matrices (Figure 1A, Supplementary Video S1). Interestingly, during growth of LECs, we noticed that they became elongated when not fed for 3 days. However, after feeding, these cells flattened back into a classic EC cobblestone morphology. We have not observed this phenomenon with BEC cultures (Supplementary Figure S1). This led us to hypothesize that LECs may be more prone to contractile behavior (perhaps due to comparatively less adherens junction components such as VE-cadherin) which may account for their inability to form and maintain tube networks. Furthermore, we thought that the LECs might be activating a pro-regressive pathway that has some relationship to our previously reported work showing that BEC tubes collapse and regress following exposure to proinflammatory mediators such as IL-1β and TNFα.

We recently described a four-drug pharmacologic cocktail known as FIST (Koller et al., 2020). This combination of forskolin, IBMX (stimulating cyclic AMP levels), SB239063 (inhibiting p38 Map kinase), and tubacin (inhibiting tubulin deacetylase HDAC6) rescued BEC capillary regression when exposed to single pro-regressive mediators, such as IL-1β and TNFα (Koller et al., 2020). Remarkably, addition of FIST with the “Factors”, dramatically stimulates LEC lumen and tube network formation in either 3D collagen or fibrin matrices (Figure 1, Supplementary Figures S3, S4, Supplementary Videos S2, S3). Real-time movies reveal marked LEC intracellular vacuolation and lumen development as well as the assembly of branched networks of tubes in 3D collagen (Supplementary Video S2) or fibrin (Supplementary Video S3) matrices. Representative cultures at 72 h were fixed and stained with anti-CD31 (green) and Hoechst (blue) and confocal z-stack pictures were obtained. These images were compiled into a single 2D “Downward” view image or were rotated 90° for a “Cross sectional” view (Figures 1B–D). In a visual z-stack reconstruction, LECs tubes are observed to possess clearly defined lumens, which are also dramatically visualized in the real-time movies (Figure 1D, Supplementary Videos S2, S3). Finally, we assessed and compared the mRNA expression of key genes between LECs and HUVECs (human umbilical vein endothelial cells) which were grown using the same culture conditions and growth media prior to performing tube morphogenesis assays. Using RT-PCR analysis, LECs showed higher mRNA expression of *PDPN* (podoplanin), *VEGFR3* (vascular endothelial growth factor receptor 3), *PROX1* (prospero homeobox 1), *NRP2* (neuropilin 2), and *TGFβ2* (transforming growth factor beta 2) and lower expression of *ICAM1* (intercellular cell adhesion molecule-1), *CD34* (cluster of differentiation 34), and *IL1R1* (interleukin 1 receptor type 1) when compared to HUVECs (Figure 1E). These expression patterns are consistent with the LEC lineage. Both types of ECs expressed mRNAs for the TNFα receptor, *TNFR1*, as well as two thrombin receptors, *PAR1* and *PAR4* (Figure 1E).

### Identification of proinflammatory mediators that directly induce lymphatic capillary tube regression

**FIGURE 2:**
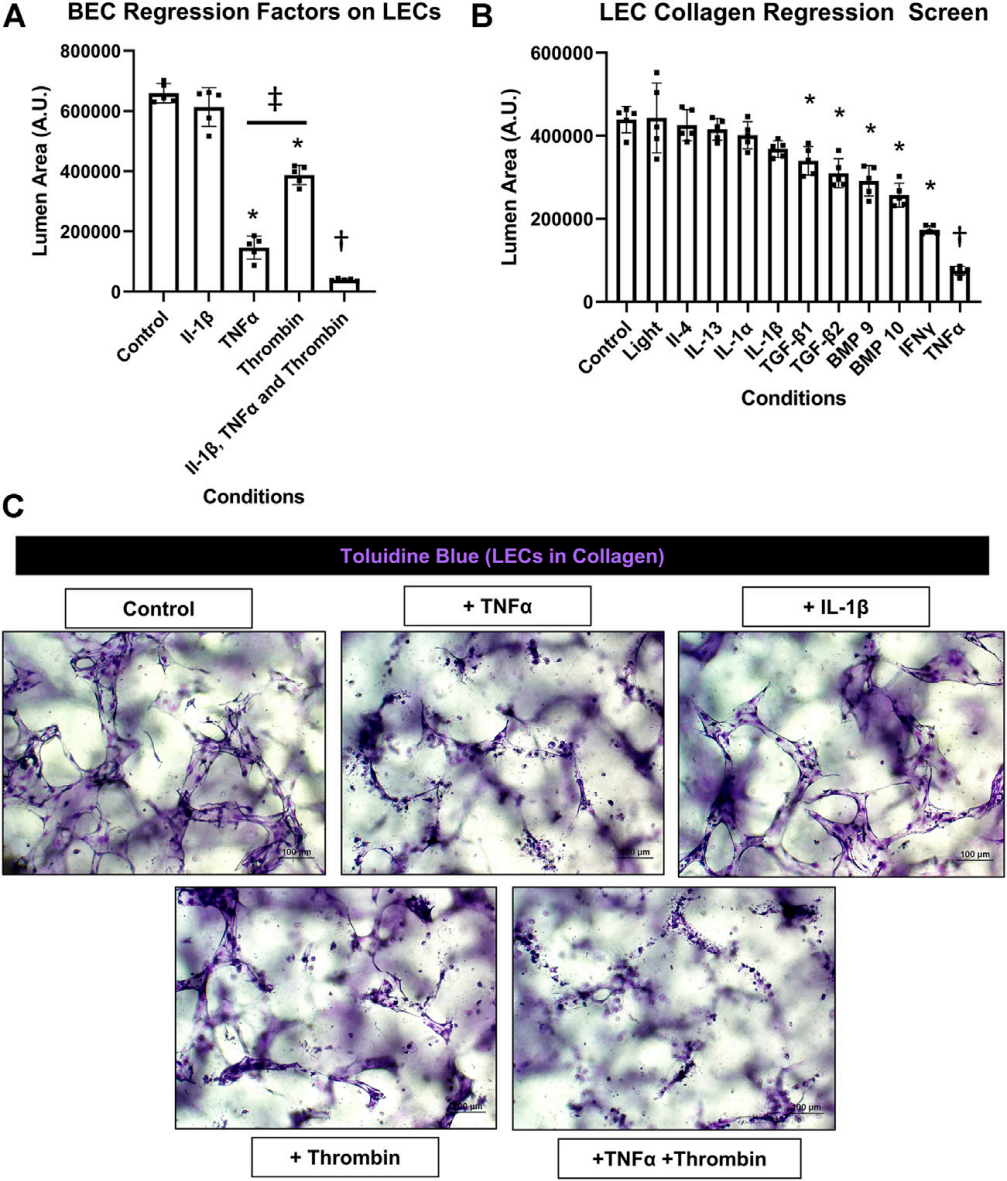
Identification of proinflammatory mediators that induce lymphatic EC tube regression in 3D collagen gels. **(A,B,C)** Serum-free lymphatic endothelial cell (LEC) tube networks + FIST (10 μM forskolin, 100 μM IBMX, 10μM SB239063, and 10 μM Tubacin) formed for 48 h in a collagen gel and were then treated with the indicated individual and combinations of growth factors and cytokines for an additional 24 h. Cultures were then fixed, stained, and photographed to evaluate EC tube area. **(A)** Blood EC (BEC) regression factors on LECs. IL (interleukin)-1β was added at 10 ng/ml, TNF (tumor necrosis factor) α was added at 10 ng/ml, thrombin was added at 1 μg/ml. **(B)** LEC collagen regression screen. Light, IL (interleukin)-4, -13, -1α, -1β, TGF (transforming growth factor)-β1 and 2, BMP (bone morphogenic protein)-9, -10, IFN (interferon) γ, TNF (tumor necrosis factor) α were all added at 10 ng/ml. **(C)** 3D LEC cultures fixed and stained with toluidine blue at the end of 72 h (48 h setup +24 h condition treatment). Bars equals 100 μm; *n* ≥ 5; *p ≤0 .05 to control, † indicates *p* ≤0.05 significance from all other conditions, ‡ indicates *p* ≤0 .05 between indicated conditions. Analyzed using one-way ANOVA with post hoc Tukey tests.

**FIGURE 3:**
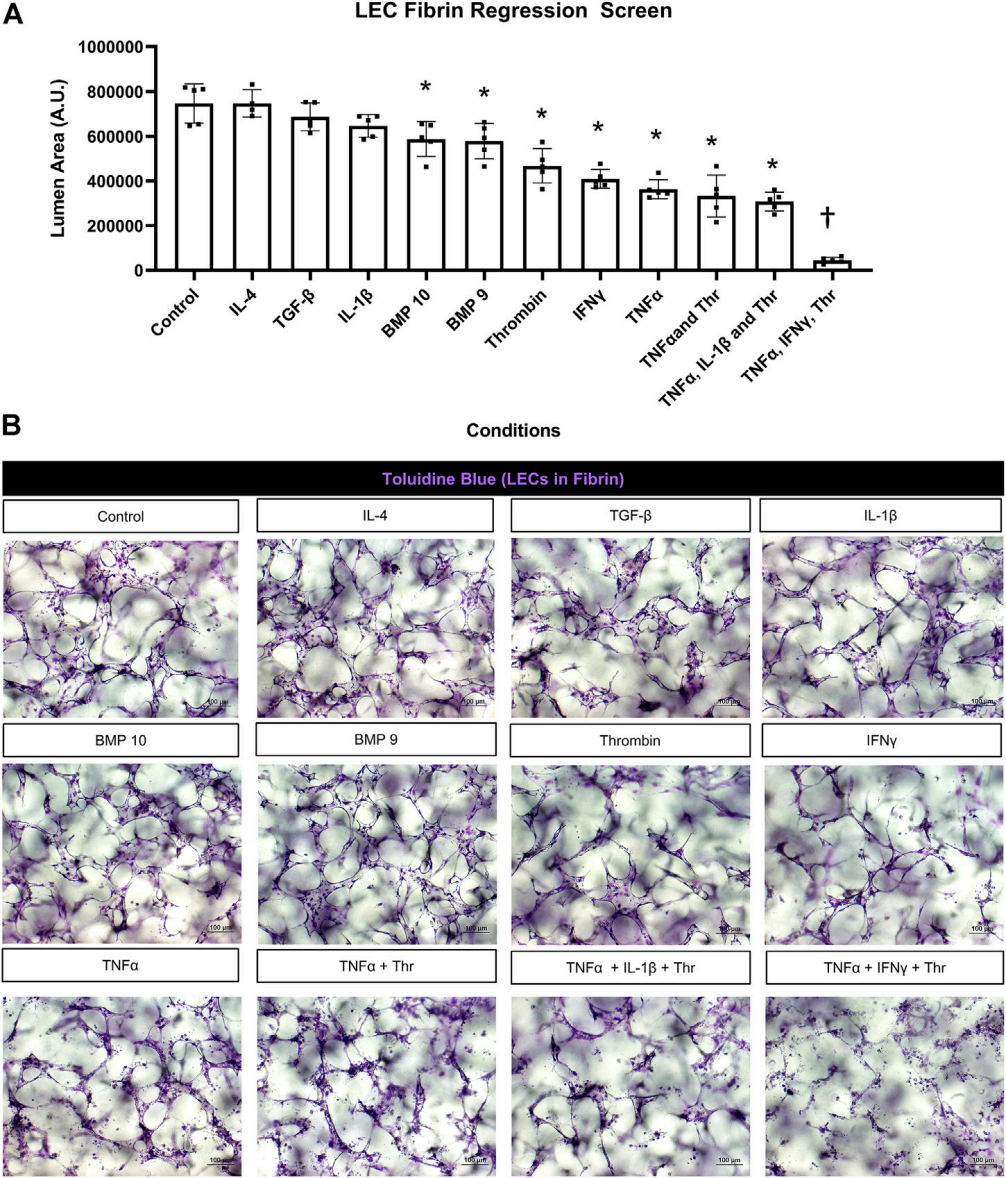
Identification of proinflammatory mediators that induce lymphatic EC tube regression in 3D fibrin gels. **(A,B)** Serum-free lymphatic endothelial cell (LEC) tube networks + FIST (10 μM forskolin, 100 μM IBMX, 10μM SB239063, and 10 μM Tubacin) were formed for 48 h in a fibrin gel and were then treated with the indicated individual and combinations of growth factors and cytokines for an additional 24 h. Cultures were then fixed, stained, and photographed to evaluate tube area. **(A)** LEC fibrin regression screen. IL (interleukin)-4 and -1β, TGF (transforming growth factor)-β1 and 2, BMP (bone morphogenic protein)-9, -10, IFN (interferon) γ, TNF (tumor necrosis factor) α were all added at 10 ng/ml; thrombin added at 1 μg/ml. **(B)** 3D LEC cultures fixed and stained with toluidine blue at the end of 72 h (48 h setup +24 h condition treatment). Bars equals 100 μm; n ≥ 5; *p ≤0.05 to control, † indicates *p* ≤0 .05 significance from all other conditions. Analyzed using one-way ANOVA with post hoc Tukey tests.

Previously, we reported that BEC-derived capillary networks were susceptible to proinflammatory mediator-induced regression (Koller et al., 2020). The most powerful inducers of regression were IL-1β and TNFα as individual factors and when combined with thrombin, even more rapid and dramatic regression occurred. Other mediators and growth factors were identified that induced BEC tube regression including IL-1α, bone morphogenic protein (BMP)-9, BMP-10, IL-4, IL-13, IFNγ, transforming growth factor (TGF)β1, TGFβ2, and Light. Here, we screened the same set of factors to assess if they also had an ability to induce regression of LEC capillary tube networks in either 3D collagen or fibrin matrices. A general schematic and timeline of our experimental approach is shown (Figure 1A). LECs were allowed to form tubes for 48 h followed by addition of the potential pro-regressive factors vs. control for 24 h. Cultures were then fixed, stained with toluidine blue, imaged and quantitated for LEC tube area (Figures 2A,3B, 3A). For the LEC collagen assay model, we initially screened IL-1β, TNFα, thrombin and their combination because of their high potency as pro-regressive mediators for BEC tube networks (Figure 2A). Of these three mediators, the most potent pro-regressive molecule was TNFα followed by thrombin, while IL-1β had no effect (Figure 2A). Combining all three factors had a strong pro-regressive effect, which most likely was due to the additive influence of TNFα and thrombin. Further screening revealed that IFNγ was the next most potent inducer of LEC tube regression after TNFα, while IL-1α, IL-1β, IL-4, IL-13, or Light showed no pro-regressive activity (Figure 2B). Other factors which showed more modest potency were BMP-9, BMP-10, TGFβ1 and TGFβ2 (Figure 2B). When TNFα, IFNγ, and thrombin were combined, marked lymphatic capillary regression occurred as demonstrated in a real-time movie (Supplementary Video S4) and compared to control lymphatic tube networks (Supplementary Video S5).

We screened most of the same mediators and growth factors in fibrin matrices using the same experimental design. In these experiments, TNFα, IFNγ, and thrombin were the three most potent pro-regressive mediators, while BMP-9 and BMP-10 had significant, but more modest pro-regressive activity (Figure 3A). In contrast, no regressive activity was observed following the addition of IL-1β, TGFβ1, or IL-4. Representative images from these different treatment conditions are shown revealing the tube degeneration associated with addition of the pro-regressive factors (Figure 3B). The combination of TNFα, IFNγ, and thrombin showed the greatest pro-regressive activity in the 24 h assay time frame (Figure 3A). Real-time movies are shown comparing the dramatic pro-regressive effect of this mediator combination (Supplementary Video S6) versus control conditions in fibrin matrices (Supplementary Video S7).

**FIGURE 4:**
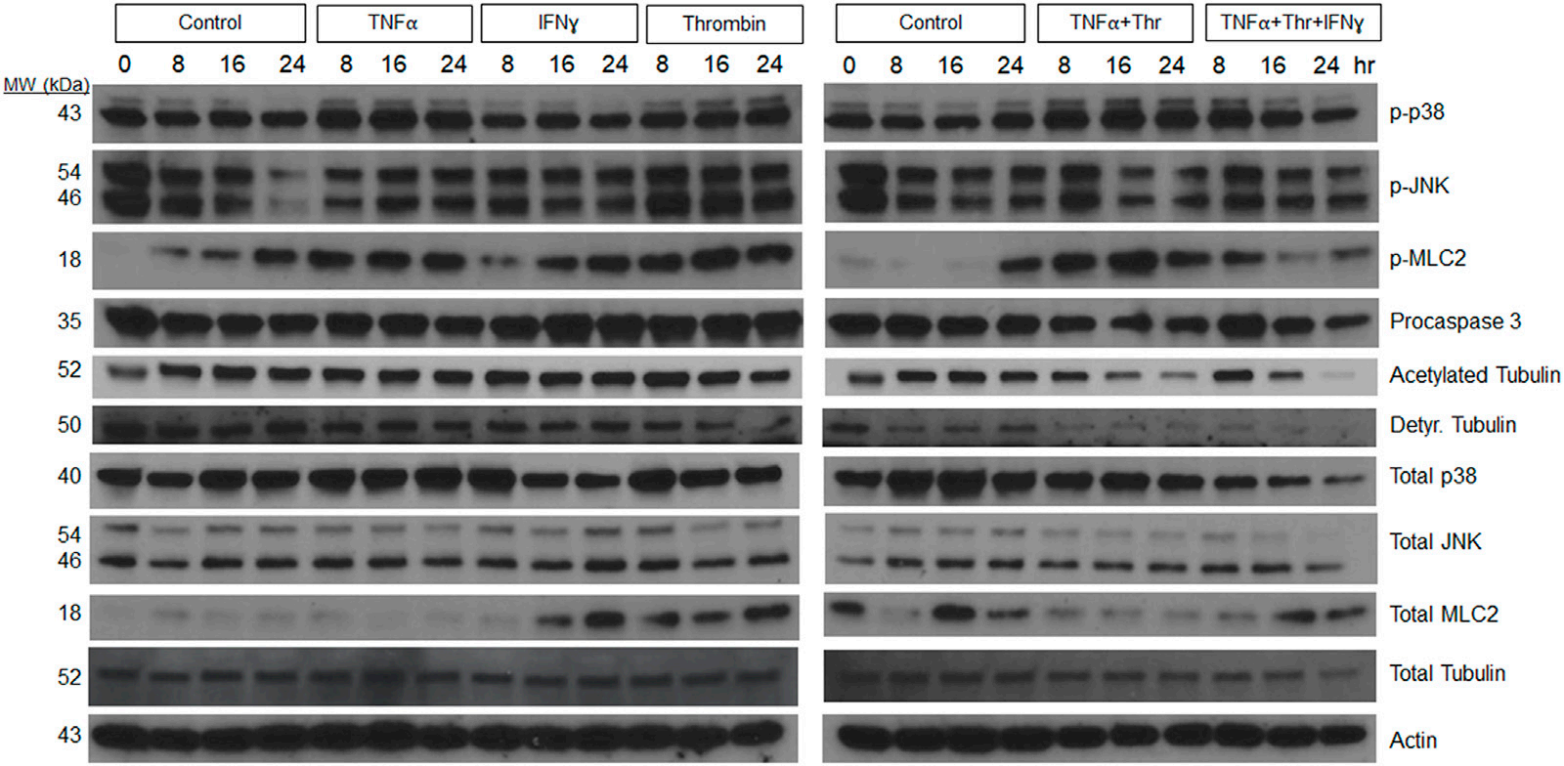
Signaling events defining lymphatic EC tube regression following exposure to TNF (tumor necrosis factor)α, IFN (interferon)γ, and thrombin (Thr). Lymphatic endothelial cell (LEC) lysates were prepared from 3-dimensional (3D) cultures at 72 h (i.e. after 24 h of exposure to individual factors or combinations of TNFα 10 ng/ml, IFNγ 10 ng/ml or Thr 1 μg/ml vs. controls). Western blots were performed and probed with the indicated antibodies including phosphorylation site targets and their control proteins. Phospho-p38 (phosphorylated p38 mitogen-activated protein kinase), phospho-JNK (phosphorylated Jun kinase), phospho-MLC2 (phosphorylated myosin light chain 2), Detyr. Tubulin (detyrosinated tubulin).

Additional experiments were performed to assess how individual, and combinations of pro-regressive mediators affected signal transduction within the LEC tube networks over a 24 h period (Figure 4). Previously, we identified a regression signaling signature for blood EC-lined tubes in response to IL-1β and TNFα that involved increased phosphorylation of p38 Map kinase (Mapk), Jun kinase (Jnk), myosin light chain (MLC) 2, and loss of tubulin acetylation and procaspase 3 (as an indicator of apoptosis) (Koller et al., 2020). Here, we show that increased p38 Mapk and pMLC2 phosphorylation is observed from LEC networks following addition of TNFα, thrombin, and TNFα + thrombin compared to control conditions (Figure 4). Modest increases in phospho-JNK were also observed at 8 h following addition of these molecules, while decreases in both detyrosinated tubulin and acetylated tubulin as well as procaspase 3 were observed during LEC regression compared to control conditions (Figure 4). Thus, there are similarities in the regression signaling events when LEC lined tubes regress compared to those composed of BECs in our previous studies (Koller et al., 2020).

### Pharmacological rescue of TNFα, IFNγ and thrombin-induced LEC tube network regression

**FIGURE 5:**
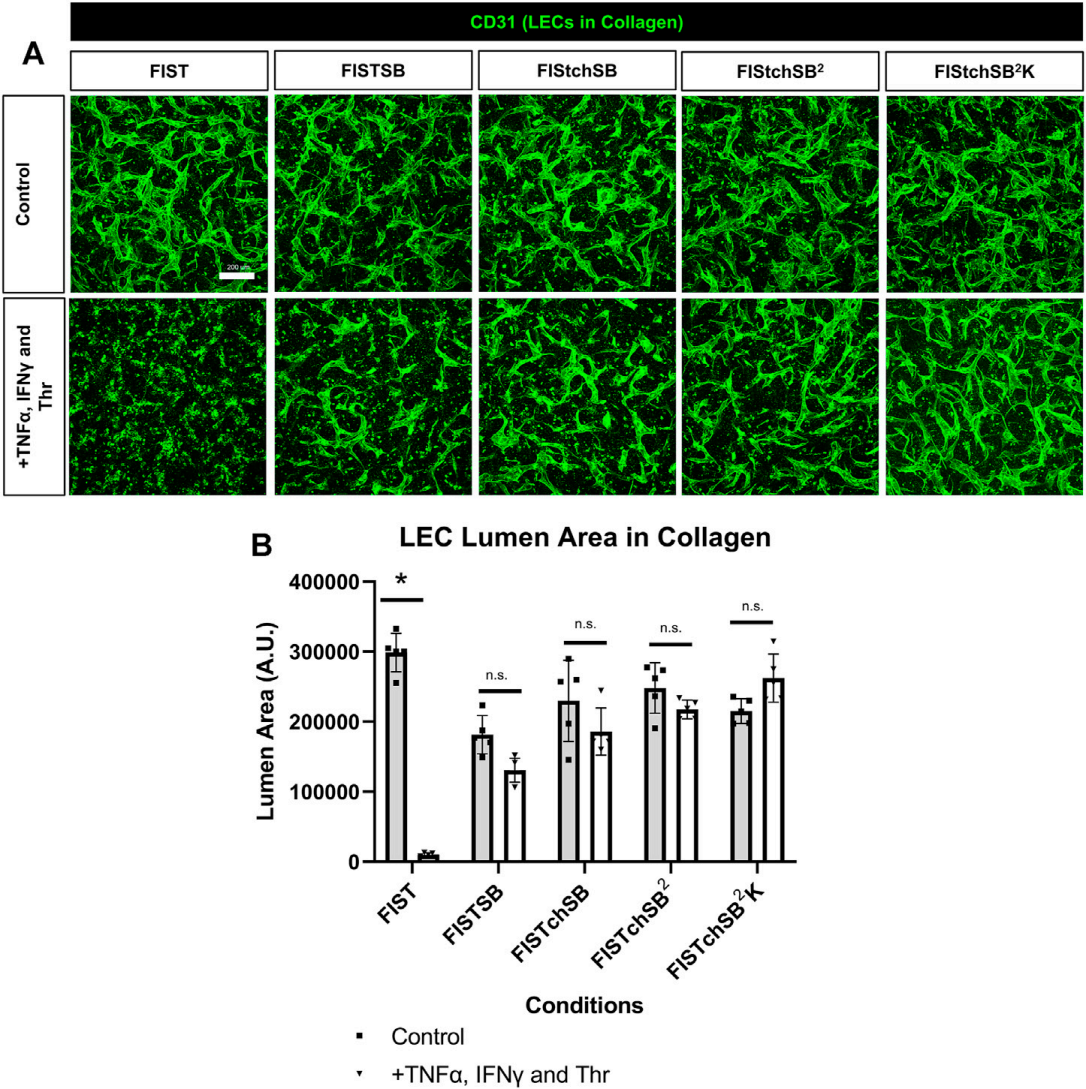
Rescuing lymphatic EC tube regression in collagen matrices induced by TNF (tumor necrosis factor) α, IFN (interferon) γ, and thrombin using combinations of pharmacologic agents. **(A,B)** Lymphatic endothelial cell (LEC) tube networks were established for 48 h using four-drug pharmacologic combination FIST (10 μM forskolin, 100 μM IBMX, 10μM SB239063, and 10 μM Tubacin) in 3-dimensional (3D) collagen gels. After 48 h, cells were treated with a combination of TNFα 10 ng/ml, IFNγ 10 ng/ml and Thr 1 μg/ml, with FIST or additional pharmacologic agents: FIST+ 25 μM SB415286 (FISTSB); FIS +10 μM TCS HDAC6 20b (tuberous sclerosis complex histone deacetylase 6) + 25 μM SB415286 (FISTchSB); FISTchSB +10 μM SB431542 (FISTchSB^2^); FISTchSB^2^ + 10 μM K 02288 (FISTchSB^2^K). **(A)** Fixed cultures were stained with anti-CD31 (green) and imaged using confocal z-stack, 3D reconstructions. Control (Top row); +TNFα, IFNγ and Thr (Bottom row); pharmacologic rescue is indicated by column; from left to right FIST, FISTSB, FISTchSB, FISTchSB^2^, FISTchSB^2^K. **(B)** After 24 h of treatment, cultures were fixed, then lumen area was evaluated. Bar equals 200 μm; *n* ≥ 5; *p ≤0 .05 compared to control condition indicated. n.s. compared to control condition indicated. Analyzed using two-way ANOVA with post hoc Tukey tests.

**FIGURE 6:**
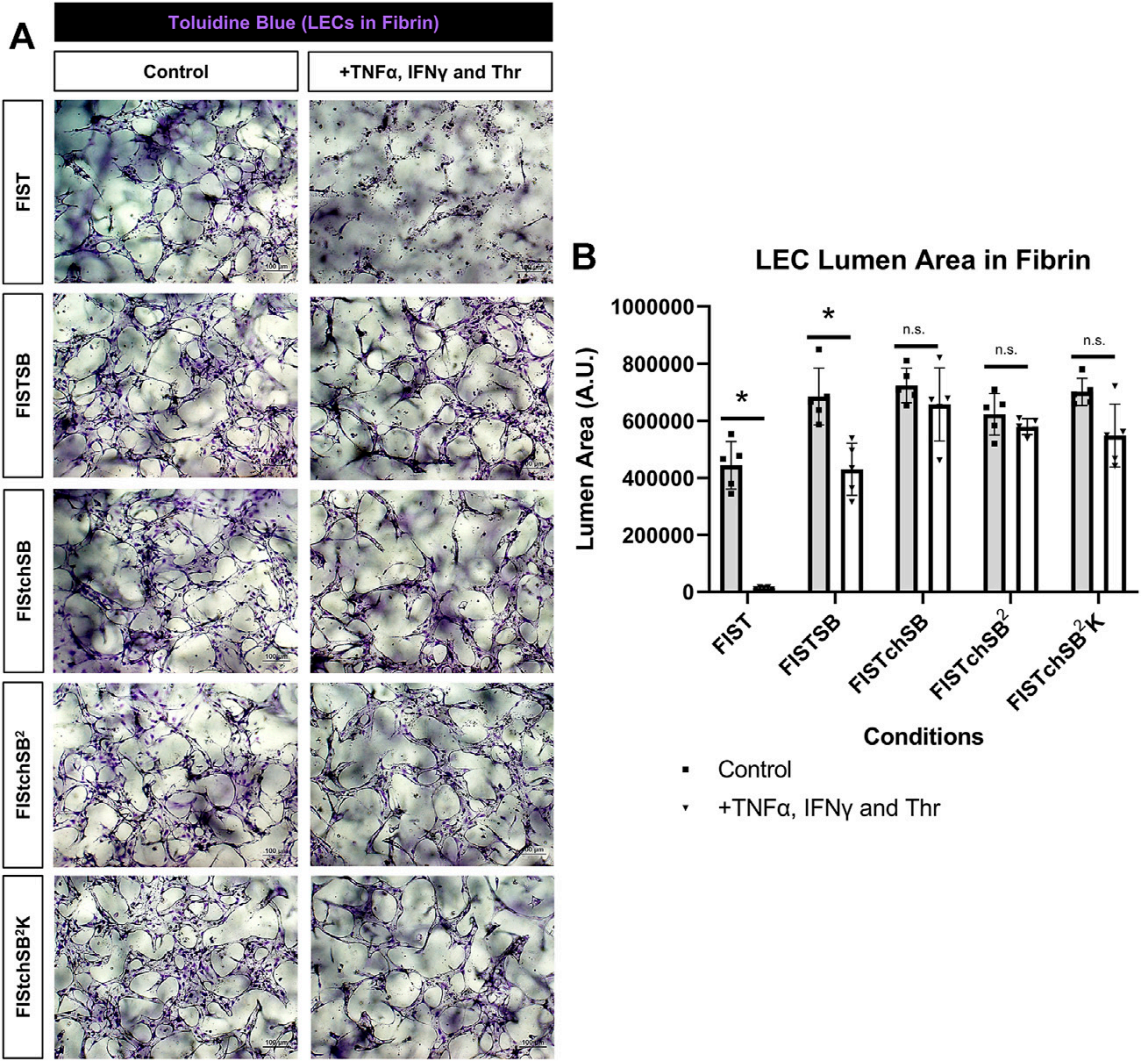
Rescuing lymphatic EC tube regression in fibrin matrices induced by TNF (tumor necrosis factor) α, IFN (interferon) γ, and thrombin using combinations of pharmacologic agents. **(A,B)** Lymphatic endothelial cell (LEC) tube networks were established for 48 h using four-drug pharmacologic combination FIST (10 μM forskolin, 100 μM IBMX, 10μM SB239063, and 10 μM Tubacin) in 3-dimensional (3D) fibrin gels. After 48 h, cells were treated with a combination of TNFα 10 ng/ml, IFNγ 10 ng/ml and Thr 1 μg/ml, with FIST or additional pharmacologic agents: FIST+ 25 μM SB415286 (FISTSB); FIS +10 μM TCS HDAC6 20b (tuberous sclerosis complex histone deacetylase 6) + 25 μM SB415286 (FISTchSB); FISTchSB +10 μM SB431542 (FISTchSB^2^); FISTchSB^2^ + 10 μM K 02288 (FISTchSB^2^K). **(A)** Fixed cultures were stained with toluidine blue and imaged. Control (**Left** column); +TNFα, IFNγ and Thr (**Right** column); pharmacologic rescue is indicated by column; from top to bottom: FIST, FISTSB, FISTchSB, FISTchSB^2^, FISTchSB^2^K. **(B)** After 24 h of treatment, cultures were fixed, then lumen area was evaluated. Bar equals 100 μm; *n* ≥ 5; *p ≤0.05 compared to control condition indicated. n.s. compared to control condition indicated. Analyzed using two-way ANOVA with post hoc Tukey tests.

**FIGURE 7:**
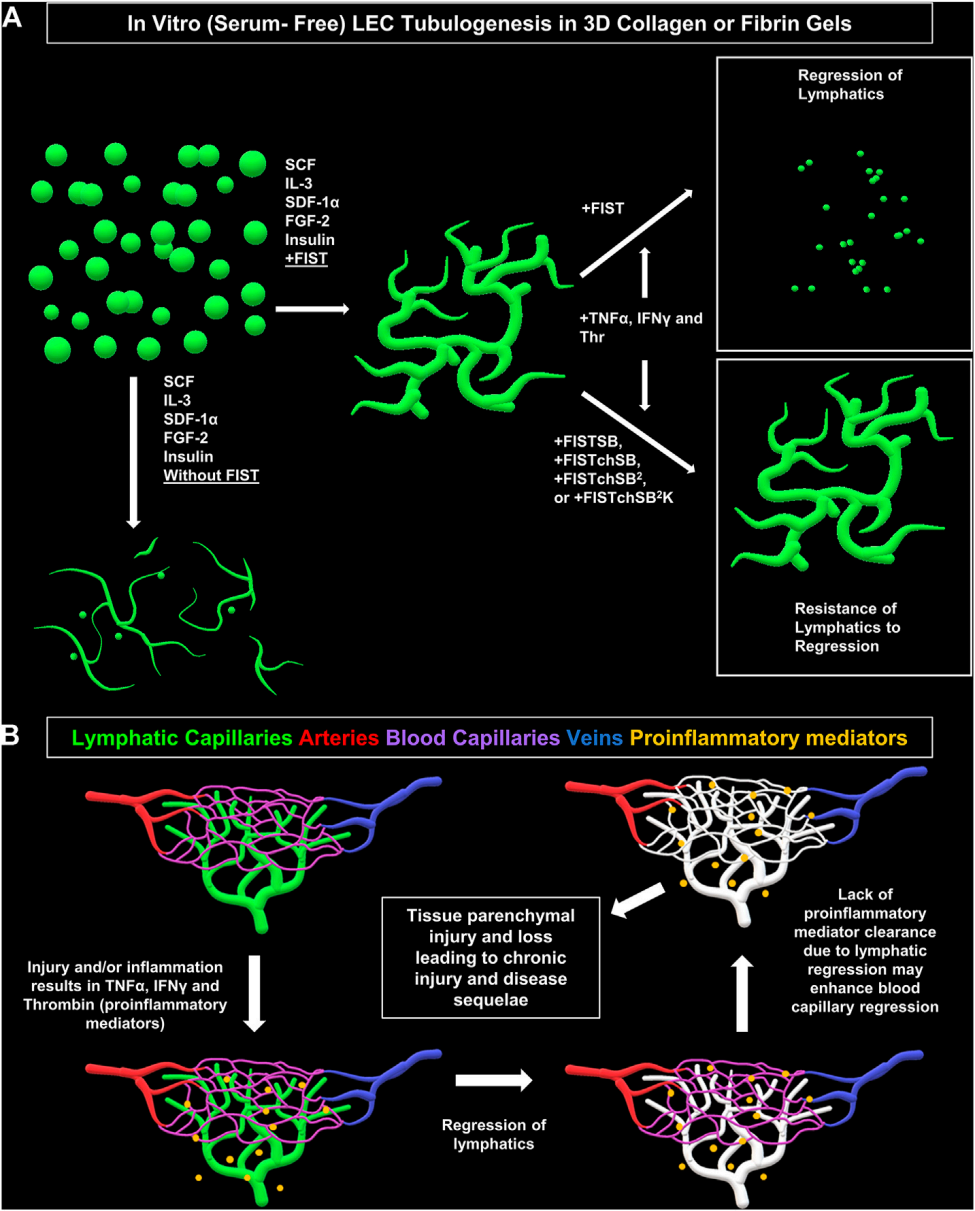
Schematic diagram illustrating formation of lymphatic EC tube network formation, proinflammatory mediators causing lymphatic EC capillary regression, as well as pharmacologic rescue of LEC when exposed to proinflammatory mediators. **(A)** Schematic illustration of our serum-free, five growth factor system (IL [interleukin]-3, SCF [stem cell factor, SDF [stromal cell-derived factor]-1α, FGF [fibroblast growth factor]-2, and insulin); along with four-drug pharmacologic combination FIST (10 μM forskolin, 100 μM IBMX, 10μM SB239063, and 10 μM Tubacin) added that induces lymphatic endothelial cells (LECs) to form tube networks in either collagen or fibrin 3D gels. TNF (tumor necrosis factor) α, IFN (interferon) γ, and thrombin cause regression of the LEC tube networks in either fibrin or collagen gels. This can be ameliorated by additional drug combinations of FIST+ 25 μM SB415286 (FISTSB); FIS +10 μM TCS HDAC6 20b (tuberous sclerosis complex histone deacetylase 6) + 25 μM SB415286 (FISTchSB); FISTchSB +10 μM SB431542 (FISTchSB^2^); FISTchSB^2^ + 10 μM K02288 (FISTchSB^2^K). **(B)** We hypothesize that LEC tube regression in response to pro-inflammatory mediators may accentuate and promote tissue injury responses due to persistence of these mediators, an effect that might further induce blood capillary regression and tissue parenchymal cell loss.

FIST was originally described as a rescue cocktail, capable of interfering with capillary regression caused by the individual mediators, TNFα, IL-1β or macrophage conditioned medium that was derived from macrophages treated with the toll-like receptor agonists, lipopolysaccharide or Pam3CSK4 (Koller et al., 2020). While the addition of FIST markedly stimulates LEC tube networks to form, it fails to rescue LEC tube networks that are exposed to additional pro-regressive agents, such as the combination of TNFα, IFNγ and thrombin (Figures 2–4). In the same paper describing blood capillary regression, a more potent rescue combination was identified, by adding an additional pharmacologic agent consisting of FIST plus SB (SB415286 [known inhibitor of GSK3β]) = FISTSB (Koller et al., 2020). Additional ongoing work has been focused on further enhancing the rescue of BEC tube network regression and has identified new pharmacological combinations capable of rescuing each of the proinflammatory mediator-induced regression responses that we have thus far observed (Penn et al., in preparation). These novel drug combinations include the base FISSB plus additional agents; +Tch (TCS HDAC6 [known HDAC6 inhibitor]) to replace tubacin = FISTchSB; +SB (SB431542 [known inhibitor of ALK5, ALK4 and ALK7] = FISTchSB^2^; +K (K02288 [known ALK2 and ALK1 receptor inhibitor]) = FISTchSB^2^K. We assessed the ability of these different drug mixtures to rescue lymphatic capillary regression in response to the potent combination of TNFα, IFNγ, and thrombin in either collagen or fibrin matrices (Figure 5 and Figure 6). Time-lapse movies were also performed to visualize these rescue effects in both collagen and fibrin systems compared to control regression responses (Supplementary Videos S4–S15). In collagen matrices, each of the drug combinations (i.e. FISTSB, FISTchSB, FISTchSB^2^, and FISTchSB^2^K) partially or fully rescued the regression response when it was compared to its treatment control (Figure 5B) (Supplementary Videos S4–S9). This conclusion is also reached by visualizing confocal images of anti-CD31 immunostains of these cultures (Figure 5A). The same result was also observed in fibrin matrices, except for the addition of FISTSB, which partially rescued regression (Figure 6B) (Supplementary Videos S10–S15). Representative toluidine blue stained images of these fibrin cultures also support these conclusions (Figure 6A). These data reveal that the FISTchSB, FISTchSB^2^ and FISTchSB^2^K drug combinations each demonstrate an equivalent ability to rescue lymphatic capillary regression from the combined influence of TNFα, IFNγ, and thrombin in either collagen or fibrin matrices (Figures 5, 6). Overall, the data presented in this work demonstrates a novel human LEC tubulogenesis assay model system which was utilized to investigate whether we could identify molecules that could promote regression of LEC tube networks. We identified that TNFα, IFNγ and thrombin showed potent abilities to promote lymphatic capillary regression and showed even greater pro-regressive effects when they were combined. Finally, we identified pharmacological agents that could rescue lymphatic capillary regression in response to these combined pro-regressive mediators (Figure 7A).

## Discussion

Our laboratory previously reported that various proinflammatory mediators induced blood capillary tube regression using serum-free defined 3D cultures (Koller et al., 2020). The most potent combination of pro-regressive factors was a mixture of IL-1β, TNFα, and thrombin (Koller et al., 2020). As lymphatics are thought to clear proinflammatory mediators and leukocytes and play a major role in the resolution of inflammation, we decided to investigate the direct effects of these and other related molecules on human lymphatic tube networks. An interesting and significant question is also to understand the dynamic relationships between BEC- and LEC-derived capillary tube networks residing in normal versus inflamed or injured tissue beds. During tissue injury events, the performance and proper functioning of these vascular networks is central in determining the consequences of the injury within specific tissue environments. To address the potential role of inflammatory mediators on lymphatic capillary networks, we first needed a robust, reproducible, serum-free, lymphatic vessel assembly system in 3D collagen or fibrin gels to evaluate in comparison to our well characterized capillary tube assembly models with BECs. To this end, we report here such a new model system using human LECs cultured in either 3D collagen or fibrin matrices. In this new system, LECs dramatically form tube networks over 48–72 h. Perhaps the most dramatic demonstration of these results are real-time movies which show this LEC morphogenesis response in either matrix environment (Supplementary Videos S2, S3). Immunostained cultures using CD31 antibodies also demonstrate the extent of this LEC tube morphogenic assembly process throughout the thickness of the 3D gels (Figure 1, Supplementary Figures S2, S3).

Using this novel LEC capillary formation platform, we screened and identified proinflammatory mediators that induce lymphatic capillary regression. Screening single factors showed that the most potent regressive agent was TNFα, followed by IFNγ and thrombin. The most severe regression response was observed when a combination of TNFα, IFNγ and thrombin was added. These mediators are generated by major proinflammatory cell types including macrophages, mast cells, neutrophils, and lymphocytes, and via enhanced permeability of the microvasculature. Interestingly, IL-1β and IL-1α had no ability to induce regression of LEC tube networks, despite their potent ability to induce regression of BEC capillary networks (Koller et al., 2020). Both IL-1β and IL-1α act through IL-1R1 to exert their biological effects. To provide a possible mechanistic reason for this difference, we observed a much lower level of *IL1R1* mRNA expression in LECs compared to BECs. Given that IL-1β is one of the most potent pathologic molecules in organisms, this could represent a defense and protective mechanism for tissue health and to dampen the severity of tissue injury responses. Thus, IL-1β may be cleared by lymphatics, keeping it from accumulating and causing destruction of tissue beds and their local blood vasculature. In fact, a recent study showed that locally generated and macrophage-derived IL-1β can stimulate the ability of mouse omental lymphatics to take up extravasated proteins, fluids, and red blood cells during the development of this tissue (Menendez et al., 2022).

In contrast to IL-1β, TNFα, and IFNγ had significant pro-regressive effects for both LEC and BEC tube networks. Thrombin also induced LEC regression and enhanced the pro-regressive effects of TNFα and IFNγ (Figures 2, 3). This latter effect of thrombin might result from its strong pro-contractile influence (i.e. RhoA and Rho kinase activation) on ECs. We also observed that BMP-9 and BMP-10 had modest pro-regressive effects on LECs. Previously, BEC tubes were found to significantly regress when exposed to IL-4, IL-13, TGF-β1, TGF-β2 and Light (Koller et al., 2020), while IL-4, IL-13, and Light had no significant regressive effect on LEC tube networks. TGF-β1 and TGF-β2 were observed to have modest, but significant LEC tube pro-regressive effects in collagen matrices, but not in fibrin matrices. The reason for this difference is not clear, but LEC tube formation in fibrin matrices appears to be more extensive than in collagen matrices, which may contribute to this distinction.

Next, we wanted to determine if we could rescue LEC tube networks that are exposed to the most potent combination of regression factors, TNFα, IFNγ and thrombin. In ongoing studies of BEC capillary regression, we have identified new pharmacological drug combinations that rescue capillary regression in response to the complex mixtures of proinflammatory mediators such as IL-1β, TNFα and thrombin and other combinations of pro-regressive molecules (Penn et al., in preparation). These drug combinations, including FISTchSB, FISTchSB^2^, and FISTchSB^2^K also strongly rescue LEC tube networks in response to TNFα, IFNγ, and thrombin (Figures 5, 6). Thus far in our investigations of both lymphatic and blood capillary tube regression, multiple pharmacologic agents are necessary together to rescue the pro-regressive influence of single or combinations of pro-regressive mediators, while individual agents fail to rescue. It is also clear that there are overlaps between mediators that induce capillary regression of these different types of EC-lined tubes, particularly with TNFα, IFNγ and thrombin. Since these mediators are elevated in many inflammatory and tissue injury conditions, it is probable, that both lymphatic and blood capillary tube networks could be negatively impacted together (Figure 7B).

Another key point is that lymphatic capillaries are believed to play a central role in the resolution of inflammation and tissue injuries (Breslin et al., 2018). Thus, if lymphatic capillaries regress, pro-inflammatory and pro-regressive mediators could remain in tissues longer, which might further potentiate the loss of both lymphatic and blood capillary tubes leading to accentuated tissue parenchymal damage (Figure 7B). Two recent studies focused on ischemic cardiac injury in mouse models showed that loss of lymphatics did not adversely affect cardiac myocyte loss or repair mechanisms (Keller et al., 2021; Harris et al., 2022). Thus, it certainly could be that different tissue types might be more impacted than others regarding the ability of lymphatic capillaries to resolve inflammation and affect tissue injury outcomes. One tissue that does appear to be sensitive to proinflammatory mediators, such as TNFα, is the intestinal tract, and lymphatic dysfunction does appear to play a pathogenic role in mouse models of inflammatory bowel disease (Czepielewski et al., 2021). Also, considerable human clinical data supports that therapeutic efficacy of antibody blockade of TNFα in Crohn’s disease (Townsend et al., 2020; Cui et al., 2021). Perhaps lymphatic capillary regression plays a pathogenic role in this disease. Another interesting area of consideration is the ability of malignant carcinomas (i.e. epithelial lineage) to invade and metastasize through lymphatic channels. Malignant tumors behave like chronic wounds with high pro-inflammatory mediator activity and persistence. Does lymphatic capillary regression occur in malignant tumors, and could this contribute to the ability of the tumor cells to enter lymphatic capillary channels and spread to local lymph nodes? Our new findings highlight the concept of lymphatic capillary regression, identify key pro-regressive mediators and pharmacological agents that prevent regression, and raise the possibility that lymphatic capillary regression, in response to exposure to the proinflammatory mediators, TNFα, IFNγ and thrombin, could be an important pathogenic feature of human disease states.

## Data Availability

The original contributions presented in the study are included in the article/Supplementary Material, further inquiries can be directed to the corresponding author.
