# Supplementary material for: Proinflammatory mediators, TNFα, IFNγ, and thrombin, directly induce lymphatic capillary tube regression
Source: Front Cell Dev Biol. 2022 Jul 19;10:937982. doi: 10.3389/fcell.2022.937982 (PMC9343954; doi:10.3389/fcell.2022.937982)
Supplement: Supplementary file 9 [file DataSheet1.PDF]

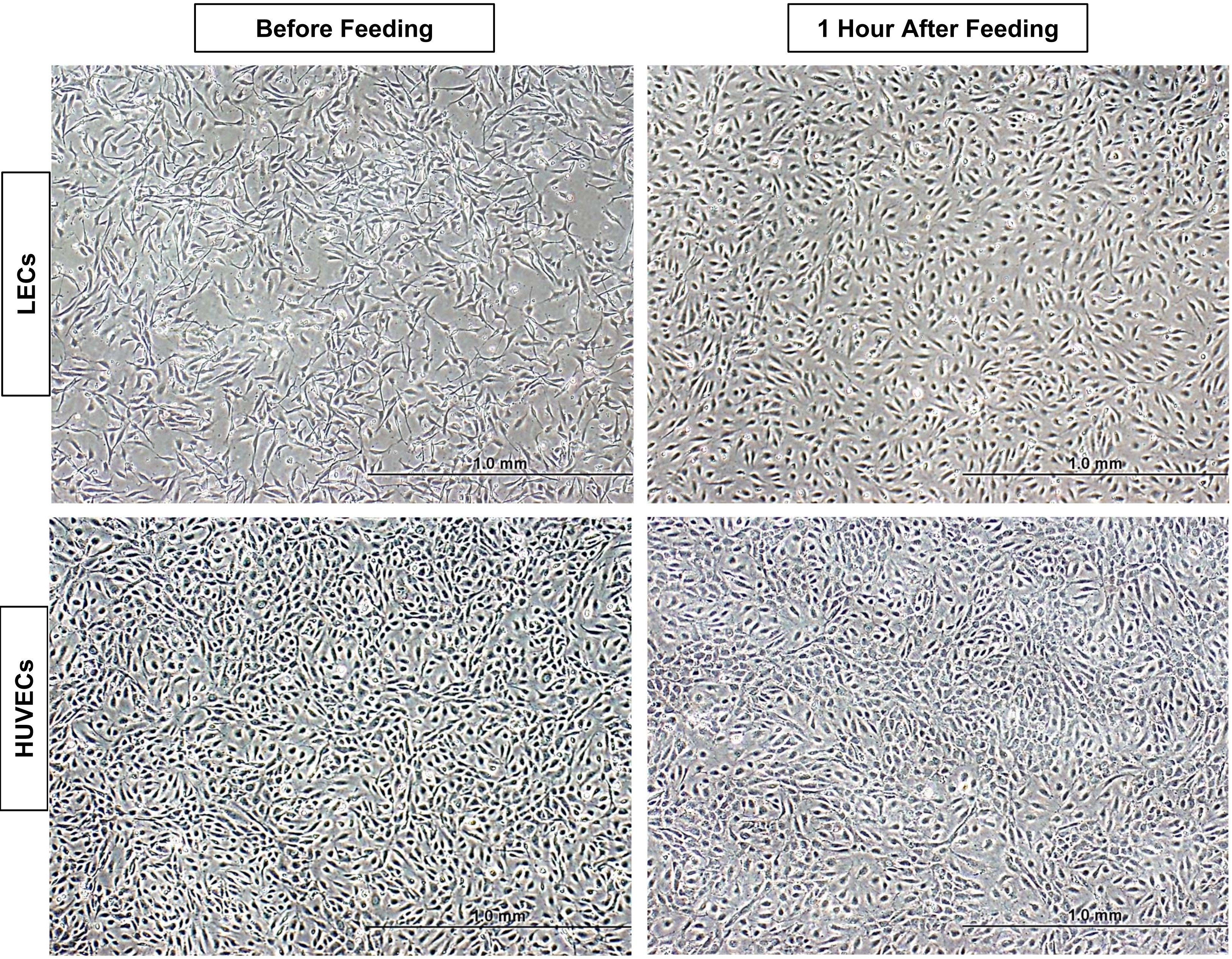

Supplemental Figure 1

CD31 (LECs in Collagen)

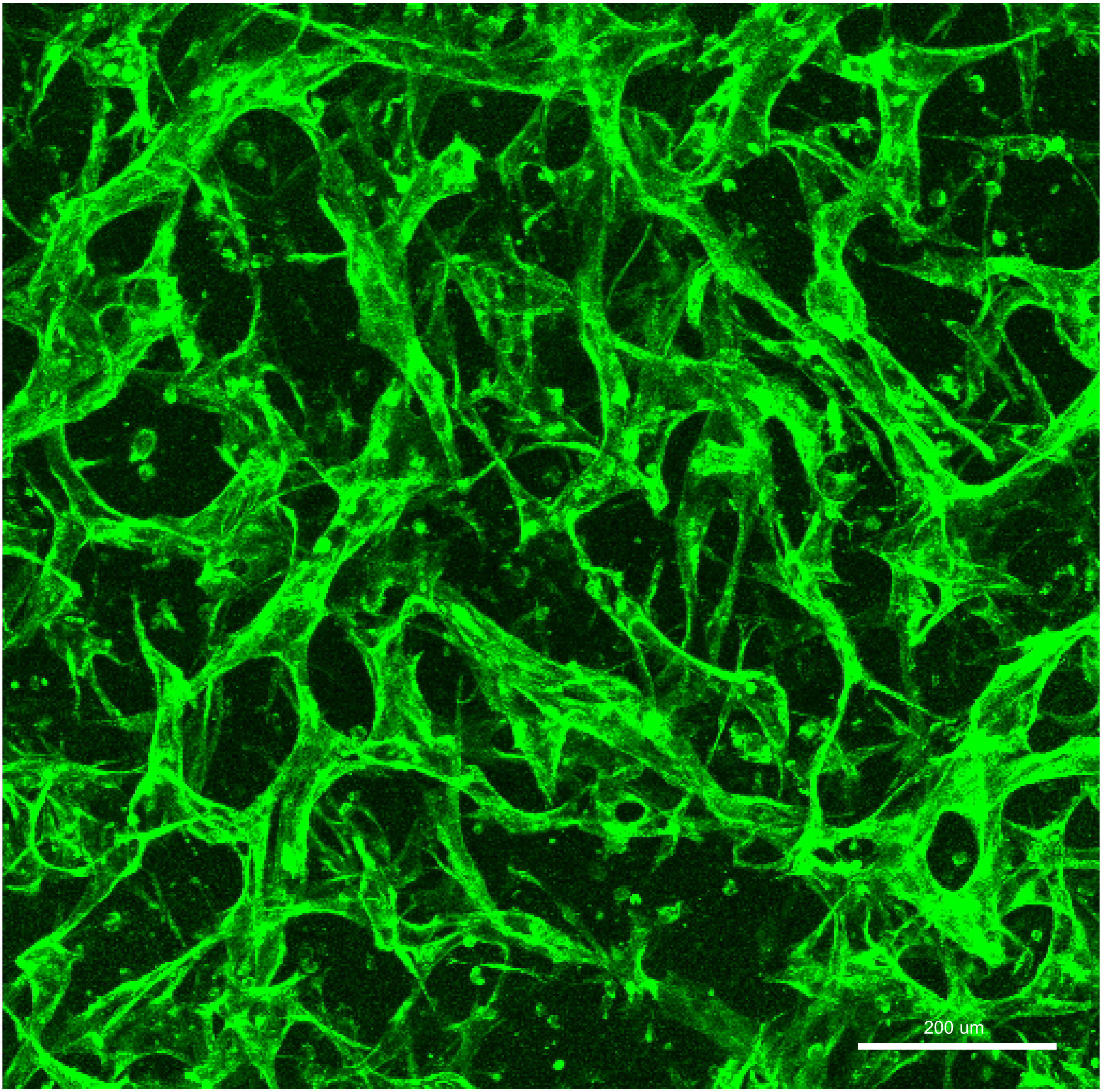

Supplemental Figure 2

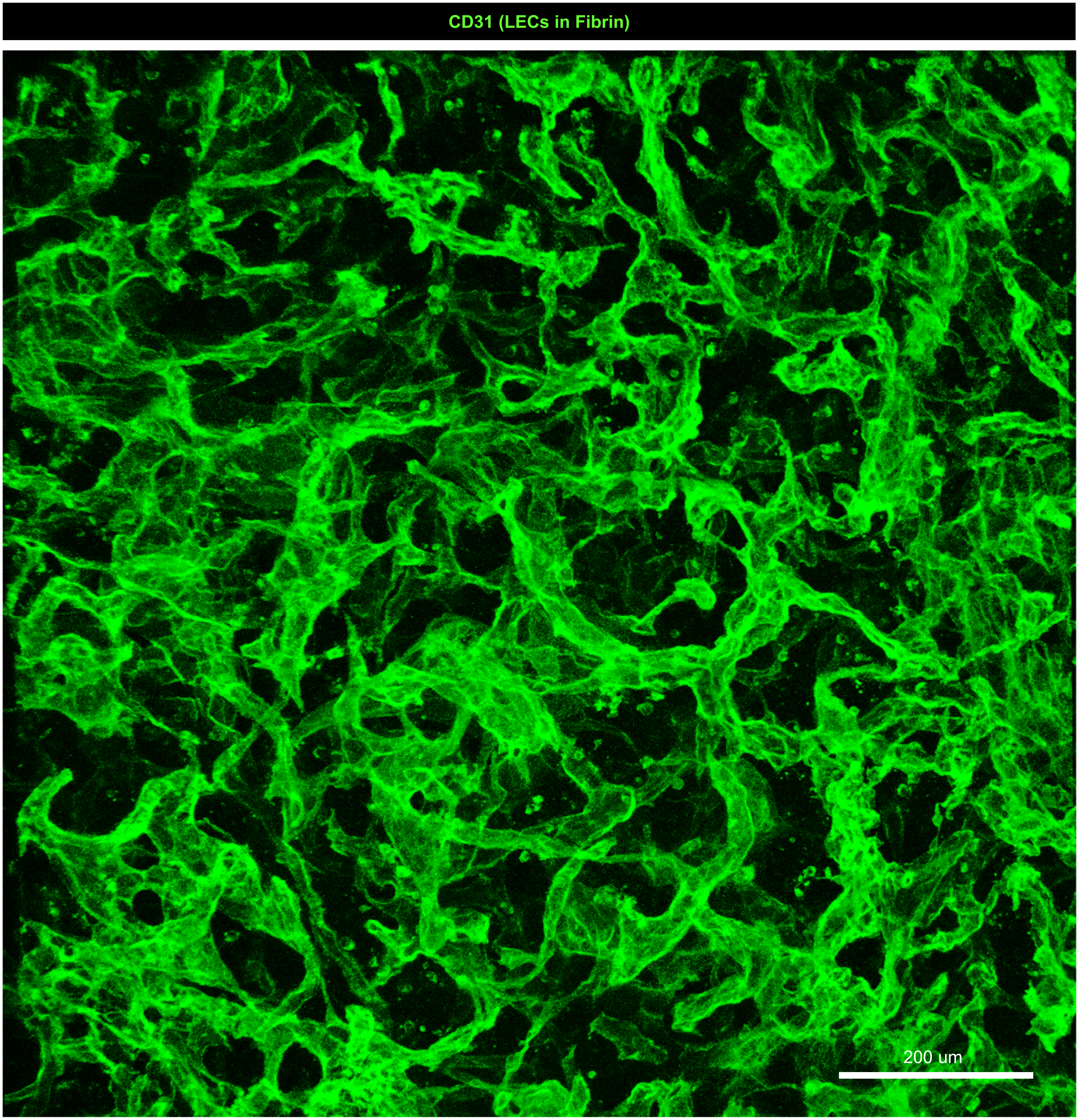

Supplemental Figure 3

Table 1. Materials and Methodological Details

Antibodies

Target antigen	Vendor or Source	Catalog #	Working concentration
CD31	Dako	GA61061-2	IF (1:200)
Goat anti-Mouse IgG, Alexa Fluor 488	ThermoFisher	A11001	IF (1:200)
Hoescht 3342	ThermoFisher	H1399	IF (1:500)
ICAM-1	R&D Systems	AF796	Western (1:1000)
p-p38	Cell Signaling	9211s	Western (1:1000)
pJNK	Cell Signaling	4668s	Western (1:1000)
p-MLC2	Cell Signaling	3674S	Western (1:1000)
Acetylated Tubulin	EMD Millipore	ABt241	Western (1:1000)
procaspase 3	Cell Signaling	9662s	Western (1:1000)
p38	Cell Signaling	9212	Western (1:1000)
JNK	Cell Signaling	9252	Western (1:1000)
MLC2	Cell Signaling	3672S	Western (1:1000)
Actin	Calbiochem	CP01	Western (1:5000)
Detyr. Tubulin	Abcam	48389	Western (1:1000)
Total Tubulin	Sigma Aldrich	T6199	Western (1:1000)

Signaling Molecules

Name	Vendor or Source	Catalog #	Working concentration
FGF2	Gibco	PHG0369	50 ng/mL
IL-3	R&D	203-IL/CF	40 ng/mL
SDF	R&D	350-NS/CF	40 ng/mL
SCF	R&D	255-SC/CF	40 ng/mL
IFNy	R&D	285-IF/CF	10 ng/mL
IL-4	R&D	204-IL/CF	10 ng/mL
IL-13	R&D	213-ILB/CF	10 ng/mL
Light	R&D	664-LI/CF	10 ng/mL
BMP-9	R&D	3209-BP/CF	10 ng/mL
BMP-10	R&D	2926-BP/CF	10 ng/mL
IL1-B	R&D	201-LB/CF	10 ng/mL
TNFa	R&D	210-TA/CF	10 ng/mL
Thrombin	EMD Millipore	604980	1 ug/mL
IL1-a	R&D	200-LA/CF	10 ng/mL
TGFB-1	R&D	240-B/CF	10 ng/mL
TGFB-2	R&D	302-B2/CF	10 ng/mL

Drugs

Drug Name	Vendor or Source	Catalog #	Working concentration
Forskolin	Tocris	1099	10μM
IBMX	Biomol	PD-140	100μM
SB239063	Tocris	1962	10μM
Tubacin	Sigma	SML0065	10μM
TCSHDAC6	Tocris	4805	10μM
SB415286	Tocris	5349	25μM
SB431542	Tocris	1614	10μM
K 02288	Tocris	4986	10μM

Other Reagents

Name	Vendor or Source	Catalog #	Working concentration
Insulin	Sigma	I2643	
Holo transferrin	Sigma	T4132	
Sodium selenite	Sigma	S1382	
BSA	Sigma	A8806	
Oleic acid	Sigma	O7501	
Dimethyl sulfoxide	Sigma	D8418	

Cultured Cells

Name	Vendor or Source	Sex (F, M, or unknown)
LECs	Lonza	Unknown

PCR Primers

Gene Name	Vendor	Forward	Reverse
GAPDH	Sigma Aldrich	AGGAGTCCACTGGCGTCTTCA	AGCAGTGAGCTTCCCGTTCAG
PROX1	Sigma Aldrich	TCCGGTTGTAAGGAGTTTGG	CAGCCCGAAAAGAACAGAAG
PDPN	Sigma Aldrich	CCAGCGAAGACCGCTATAAG	TTTTCGCATAACCACAACGA
VEGFR3	Sigma Aldrich	GCTCAAGGAGGTGACAGAGG	ACAAACTCGGTCCAGGTGTC
NRP2	Sigma Aldrich	CACTCTGTGGCCTCCTCTTC	TTGTGCGCAAGTTCAAAGTC
VEGFR2	Sigma Aldrich	AGGTGCTTCTACCGGGAAAC	AGTTCAATTCCATGAGACGG
VE-CAD	Sigma Aldrich	CCCATGAAGCCTCTGGATTATG	CAGTGGAATCCAGTTCTTTGGC
ICAM-1	Sigma Aldrich	AGTGCGGCACGAGAAATTGG	CTGTGACCAGCCCAAGTTGTTG
CD34	Sigma Aldrich	TCCTTCTTAAACTCCGCACAGC	TGCACCCTGTGTCTCAACATGG
TNFR1	Sigma Aldrich	ACCAAGTGCCACAAAGGAAC	TCTGGGGTAGGCACAACTTC

IL1R1	Sigma Aldrich	GGAGGACTTGTGTGCCCTTA	TGTTTGCAGGATTTTCCACA
PAR1	Sigma Aldrich	CTCCACTTGGCAGCTTCTTC	CAAATCTGCCGTGTATGTGG
PAR4	Sigma Aldrich	GACTCTGCATGGCTGCTTG	GCACGTAGGCACCATAGAGG
ALK5	Sigma Aldrich	AACGTCAGGTTCTGGCTCAGG	CATGTGAAGATGGGCAAGACCG
TGFβ1	Sigma Aldrich	AGGCGTGTCCAGGCTCCAAATGTAGG	AGCAGGGATAACACACTGCAAGTGGACATC
TGFβ2	Sigma Aldrich	AGAGTGCCTGAACAACGGATTG	TGGACGTAGGCAGCAATTATCC

<u>Supplemental Materials</u>

<u>Supplemental Figure Legends</u>

<u>Supplemental Figure 1:</u> **Lymphatics ECs have a notably different morphology before and after feeding on plastic surfaces, while HUVECs do not exhibit the same morphologic changes.** Representative images of lymphatic endothelial cells (LECs) after not being fed for 3 days on plastic surfaces (**top left**) or 1 hr after feeding (**top right**). Representative images of HUVECS after not being fed for 3 days on plastic surfaces (**bottom left)** or 1 hour after feeding (**bottom right**). Bar equals 1 mm.

<u>Supplemental Figure 2.</u> **Lymphatic EC tube networks stained with anti-CD31, in a 3D collagen gel after 72 hr with FIST.** Serum-free, five growth factor system (IL [interleukin]-3, SCF [stem cell factor, SDF [stromal cell-derived factor]-1α, FGF [fibroblast growth factor]-2, and insulin) with four-drug pharmacological combination FIST (10μM forskolin, 100μM IBMX, 10μM SB239063, and 10μM Tubacin) which allows lymphatic endothelial cells (LECs) to form 3-dimensional (3D) tube networks in collagen gels. LEC tube networks were fixed at 72 hr. Fixed cultures were stained with anti-CD31 (green). Imaged using confocal z-stack, 3D reconstructions. Bar equals 200 μm.

<u>Supplemental Figure 3.</u> **Lymphatic EC tube networks stained with anti-CD31, in a 3D fibrin gel after 72 hr with FIST.** Serum-free, five growth factor system (IL [interleukin]-3, SCF [stem cell factor, SDF [stromal cell-derived factor]-1α, FGF [fibroblast growth factor]-2, and insulin) with four-drug pharmacological combination FIST (10μM forskolin, 100μM IBMX, 10μM SB239063, and 10μM Tubacin) which allows lymphatic endothelial cells (LECs) to form 3-dimensional (3D) tube networks in fibrin gels. LEC tube networks were fixed at 72 hr. Fixed cultures were stained with anti-CD31 (green). Imaged using confocal z-stack, 3D reconstructions. Bar equals 200 μm.

<u>Supplemental Video Legends</u>

<u>Video 1:</u> LEC formation in fibrin matrices without FIST. 0-48 hr LEC assay in 3D fibrin gels, serum-free. The video is shown at 12 frames/sec.

Video 2: **LEC formation in collagen matrices with FIST.** 0-48 hr LEC assay in 3D collagen gels, serum-free + FIST (10μM forskolin, 100μM IBMX, 10μM SB239063, and 10μM Tubacin). The video is shown at 12 frames/sec.

Video 3: **LEC formation in collagen matrices with FIST.** 0-48 hr LEC assay in 3D fibrin gels, serum-free + FIST (10μM forskolin, 100μM IBMX, 10μM SB239063, and 10μM Tubacin). The video is shown at 12 frames/sec.

Video 4: **LECs in collagen matrices – Control FIST.** 48-72 hr LEC assay in 3D collagen gels, serum-free + FIST (10μM forskolin, 100μM IBMX, 10μM SB239063, and 10μM Tubacin). The video is shown at 12 frames/sec.

Video 5: **LECs in collagen matrices – Control FIST + Regression factors (TNFα, IFNγ and Thrombin).** 48-72 hr LEC assay in 3D collagen gels, serum-free + FIST (10μM forskolin, 100μM IBMX, 10μM SB239063, and 10μM Tubacin) with regression factors (10 ng/mL TNFα, 10 ng/mL IFNγ and 1μg/mL thrombin). The video is shown at 12 frames/sec.

Video 6: **LECs in collagen matrices – FISTSB + Regression factors (TNFα, IFNγ and Thrombin).** 48-72 hr LEC assay in 3D collagen gels, serum-free + FISTSB (10μM forskolin, 100μM IBMX, 10μM SB239063, 10μM Tubacin, and 25μM SB415286) with regression factors (10 ng/mL TNFα, 10 ng/mL IFNγ and 1μg/mL thrombin). The video is shown at 12 frames/sec.

Video 7: **LECs in collagen matrices – FISTchSB + Regression factors (TNFα, IFNγ and Thrombin).** 48-72 hr LEC assay in 3D collagen gels, serum-free + FISTchSB (10μM forskolin, 100μM IBMX, 10μM SB239063, 10μM TCS HDAC6 20b, and 25μM SB415286) with regression factors (10 ng/mL TNFα, 10 ng/mL IFNγ and 1μg/mL thrombin). The video is shown at 12 frames/sec.

Video 8: **LECs in collagen matrices – FISTchSB^2 + Regression factors (TNFα, IFNγ and Thrombin).** 48-72 hr LEC assay in 3D collagen gels, serum-free + FISTchSB^2 (10μM forskolin, 100μM IBMX, 10μM SB239063, 10μM TCS HDAC6 20b, 25μM SB415286, and 10μM SB431542) with regression factors (10 ng/mL TNFα, 10 ng/mL IFNγ and 1μg/mL thrombin). The video is shown at 12 frames/sec.

<u>Video 9:</u> LECs in collagen matrices – FISTchSB^{2}K + Regression factors (TNFα, IFNγ and Thrombin). 48-72 hr LEC assay in 3D collagen gels, serum-free + FISTchSB^{2}K (10μM forskolin, 100μM IBMX, 10μM SB239063, 10μM TCS HDAC6 20b, 25μM SB415286, 10μM SB431542, and 10μM K02288) with regression factors (10 ng/mL TNFα, 10 ng/mL IFNγ and 1μg/mL thrombin). The video is shown at 12 frames/sec.

<u>Video 10:</u> LECs in fibrin matrices – Control FIST. 48-72 hr LEC assay in 3D fibrin gels, serum-free + FIST (10μM forskolin, 100μM IBMX, 10μM SB239063, and 10μM Tubacin). The video is shown at 12 frames/sec.

<u>Video 11:</u> LECs in fibrin matrices – Control FIST + Regression factors (TNFα, IFNγ and Thrombin). 48-72 hr LEC assay in 3D fibrin gels, serum-free + FIST (10μM forskolin, 100μM IBMX, 10μM SB239063, and 10μM Tubacin) with regression factors (10 ng/mL TNFα, 10 ng/mL IFNγ and 1μg/mL thrombin). The video is shown at 12 frames/sec.

<u>Video 12:</u> LECs in fibrin matrices – FISTSB + Regression factors (TNFα, IFNγ and Thrombin). 48-72 hr LEC assay in 3D fibrin gels, serum-free + FISTSB (10μM forskolin, 100μM IBMX, 10μM SB239063, 10μM Tubacin, and 25μM SB415286) with regression factors (10 ng/mL TNFα, 10 ng/mL IFNγ and 1μg/mL thrombin). The video is shown at 12 frames/sec.

<u>Video 13:</u> LECs in fibrin matrices – FISTchSB + Regression factors (TNFα, IFNγ and Thrombin). 48-72 hr LEC assay in 3D fibrin gels, serum-free + FISTchSB (10μM forskolin, 100μM IBMX, 10μM SB239063, 10μM TCS HDAC6 20b, and 25μM SB415286) with regression factors (10 ng/mL TNFα, 10 ng/mL IFNγ and 1μg/mL thrombin). The video is shown at 12 frames/sec.

<u>Video 14:</u> LECs in fibrin matrices – FISTchSB^2 + Regression factors (TNFα, IFNγ and Thrombin). 48-72 hr LEC assay in 3D fibrin gels, serum-free + FISTchSB^2 (10μM forskolin, 100μM IBMX, 10μM SB239063, 10μM TCS HDAC6 20b, 25μM SB415286, and 10μM SB431542) with regression factors (10 ng/mL TNFα, 10 ng/mL IFNγ and 1μg/mL thrombin). The video is shown at 12 frames/sec.

__**Video 15:**__ **LECs in fibrin matrices – FISTchSB^{2}K + Regression factors (TNFα, IFNγ and Thrombin).** 48-72 hr LEC assay in 3D fibrin gels, serum-free + FISTchSB^{2}K (10μM forskolin, 100μM IBMX, 10μM SB239063, 10μM TCS HDAC6 20b, 25μM SB415286, 10μM SB431542, and 10μM K02288) with regression factors (10 ng/mL TNFα, 10 ng/mL IFNγ and 1μg/mL thrombin). The video is shown at 12 frames/sec.
